# Epigenetic signatures differentiate uterine and soft tissue leiomyosarcoma

**DOI:** 10.18632/oncotarget.28032

**Published:** 2021-08-03

**Authors:** Nesrin M. Hasan, Anup Sharma, Nensi M. Ruzgar, Hari Deshpande, Kelly Olino, Sajid Khan, Nita Ahuja

**Affiliations:** ^1^Department of Surgery, Yale University School of Medicine, New Haven, CT, USA; ^2^Yale University School of Medicine, New Haven, CT, USA; ^3^Department of Internal Medicine, Section of Medical Oncology, Yale University School of Medicine, New Haven, CT, USA; ^4^Department of Surgery, Section of Hepatopancreatobiliary and Mixed Tumors, Yale University School of Medicine, New Haven, CT, USA

**Keywords:** leiomyosarcoma, epigenetics, DNA methylation, gene expression, uterine leiomyosarcoma

## Abstract

Leiomyosarcomas (LMS) are diverse, rare, and aggressive mesenchymal soft tissue sarcomas. Epigenetic alterations influence multiple aspects of cancer, however epigenetic profiling of LMS has been limited. The goal of this study was to delineate the molecular landscape of LMS for subtype-specific differences (uterine LMS (ULMS) vs soft tissue LMS (STLMS)) based on integrated analysis of DNA methylation and gene expression to identify potential targets for therapeutic intervention and diagnosis. We identified differentially methylated and differentially expressed genes associated with ULMS and STLMS using DNA methylation and RNA-seq data from primary tumors. Two main clusters were identified through unsupervised hierarchical clustering: ULMS-enriched cluster and STLMS-enriched cluster. The integrated analysis demonstrated 34 genes associated with hypermethylation of the promoter CpG islands and downregulation of gene expression in ULMS or STLMS. In summary, these results indicate that differential DNA methylation and gene expression patterns are associated with ULMS and STLMS. Further studies are needed to delineate the contribution of epigenetic regulation to LMS subtype-specific gene expression and determine the roles of the differentially methylated and differentially expressed genes as potential therapeutic targets or biomarkers.

## INTRODUCTION

Leiomyosarcomas (LMS) are aggressive heterogeneous mesenchymal neoplasms that account for 10–20% of soft tissue sarcomas [1]. LMS arise from the smooth muscle cells of different structures and organs including the uterus, retroperitoneum, abdomen, large and medium blood vessels, trunk, and extremities [1]. Newly diagnosed patients are at high risk of distant recurrence and poor disease-specific survival [2]. The 5-year survival rate is 42% for all stages and only 14% with distant spread, based on data from the Surveillance, Epidemiology, and End Results Program (SEER) datasets. Recurrence and/or metastasis occurs in ~40% of the cases [3], limiting the treatment options to standard chemotherapy [1]; however, response to first-line systemic chemotherapy is low, ranging from 5% to 33% [4–8]. Current treatments for metastatic disease (doxorubicin, trabectidin, dacarbazine, pazopanib) are either traditional chemotherapies or a targeted tyrosine kinase inhibitor [9]. The approval of tazemetostat for patients with metastatic epithelioid sarcomas has been the first time an epigenetic modifier was shown to have a role in the treatment of soft tissue sarcomas [10]. There is little data regarding factors that influence survival in LMS patients underscoring the need for studies to understand the genetic and epigenetic factors involved in LMS which influence treatment response.

Based on the site of origin, LMS subtypes have been described including uterine LMS (ULMS) and non-uterine soft tissue LMS (STLMS) [1]. Only a limited number of studies have compared the outcomes based on LMS subtypes despite obvious clinical differences. Based on the limited literature, patients with ULMS often present with larger tumors, metastatic disease, and worse overall survival [7]. Despite this, other studies have shown that the site of origin of LMS has no impact on outcomes [11, 12]. Histologically ULMS and STLMS appear similar and share distinct features of the smooth muscle lineage with no available diagnostic or prognostic biomarkers to inform clinical management [1]. Furthermore, as both subtypes are treated similarly, studies are needed to understand if molecular profiling and subtype-specific therapeutic targeting can improve survival.

Prior studies have made limited attempts to subtype LMS using exome-based, gene expression microarray-based, or RNA-seq-based profiling approaches [13–19]. However, only few studies have focused on characterizing the epigenetic profiles of LMS and investigating the role of epigenetic changes in LMS subtype progression [18, 20, 21]. In oncogenesis, gene expression can be regulated epigenetically through changes in DNA methylation, histone modifications, and chromatin structure [22]. Methylation changes include hypomethylation of intergenic regions and hypermethylation of CpG islands along with upstream methylation of shores and shelves resulting in transcriptional changes [23, 24]. Promoter hypermethylation of p16 tumor suppressor protein and death-associated protein kinase (DAP kinase) has been associated with decreased protein expression in STLMS [25, 26]. Epigenetic regulators (HIST3H3, SETD7, KMT2C) are implicated in driving LMS tumor mutational heterogeneity [13]. Furthermore, epigenetic therapies may be promising candidates for managing sarcomas and other tumor types [27–29]. A previous study in our laboratory demonstrated the potential therapeutic application of hypomethylating agents such as DNA methyltransferase inhibitors (DNMTi) in *in vitro* and *in vivo* models of LMS [28]. These findings suggest the need for detailed epigenetic profiling of LMS tumors, exploring the roles of epigenetic alterations in oncogenesis and investigating the utility of epigenetics-based therapeutic targeting.

In this study, we performed an integrated analysis of 98 clinically derived LMS samples and 11 controls using three publicly available datasets to identify epigenetic changes which characterize LMS subtypes and may be used as clinical biomarkers and therapeutic targets. The Cancer Genome Atlas-Sarcoma (TCGA-SARC) dataset (*n* = 80, 27 ULMS and 53 STLMS) [18] was used to comprehensively understand the epigenetic and transcriptomic differences between ULMS and STLMS to identify genes that are differentially methylated and differentially expressed in different LMS subtypes. These findings were compared to two independent DNA methylation datasets, GSE140686 (*n* = 24 samples, 8 controls and 16 STLMS) [20] and GSE68312 (*n* = 5 samples, 3 controls and 2 ULMS) [30] to identify the epigenetic changes that are associated with tumorigenesis compared to controls.

Our findings reveal that LMS tumors are associated with distinct epigenetic alterations and gene expression changes. Unsupervised hierarchical clustering analysis demonstrated that LMS tumor samples are associated with two main clusters: ULMS-enriched cluster and STLMS-enriched cluster, suggesting that distinct DNA methylation changes and gene expression changes underlie the biology of each cluster. Furthermore, genes with an inverse correlation between promoter CpG island hypermethylation and gene expression were identified suggesting the epigenetic regulation of the gene expression.

## RESULTS

### ULMS and STLMS are epigenetically distinct tumor subtypes

**Figure 1:**
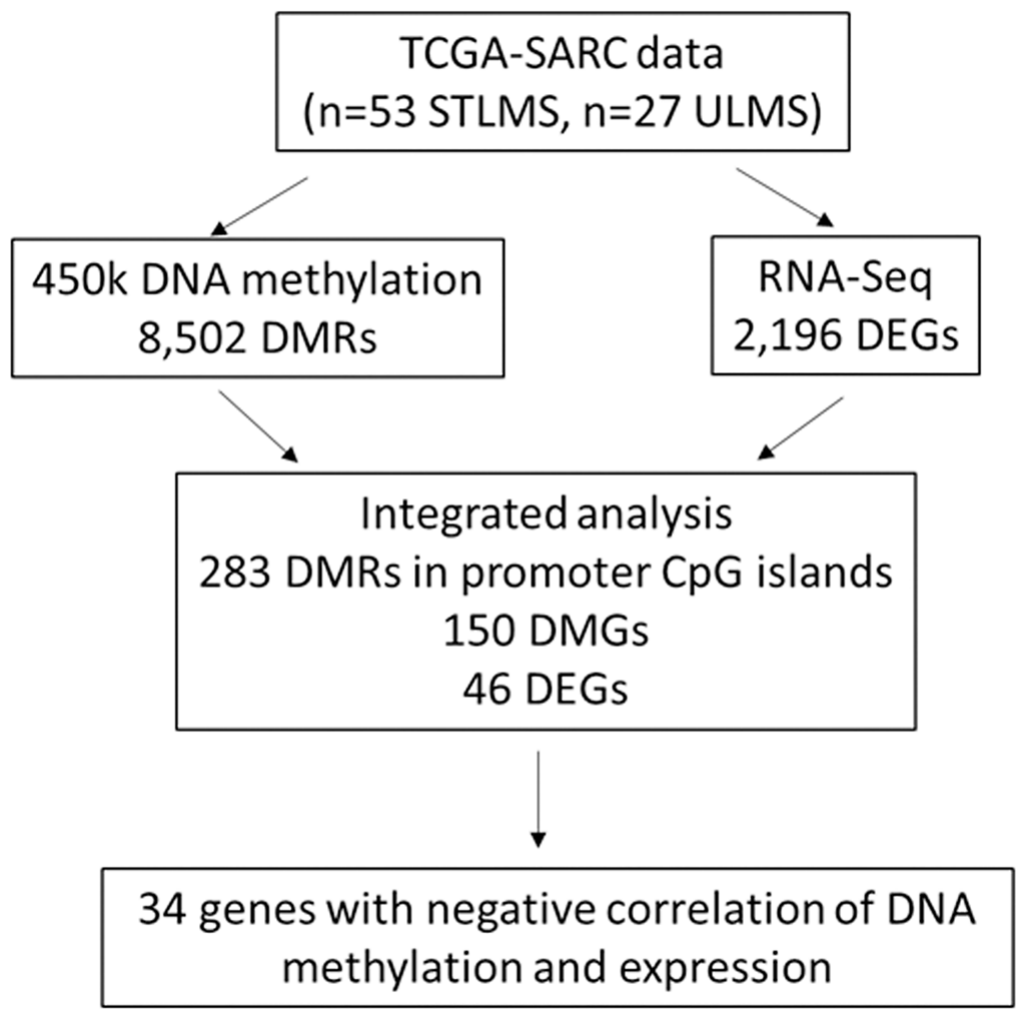
Overview of the analysis. Leiomyosarcoma (LMS) samples from the TCGA-SARC dataset were compared to identify the differentially methylated regions (DMRs), differentially methylated genes (DMGs) and differentially expressed genes (DEGs) in ULMS and STLMS.

**Figure 2:**
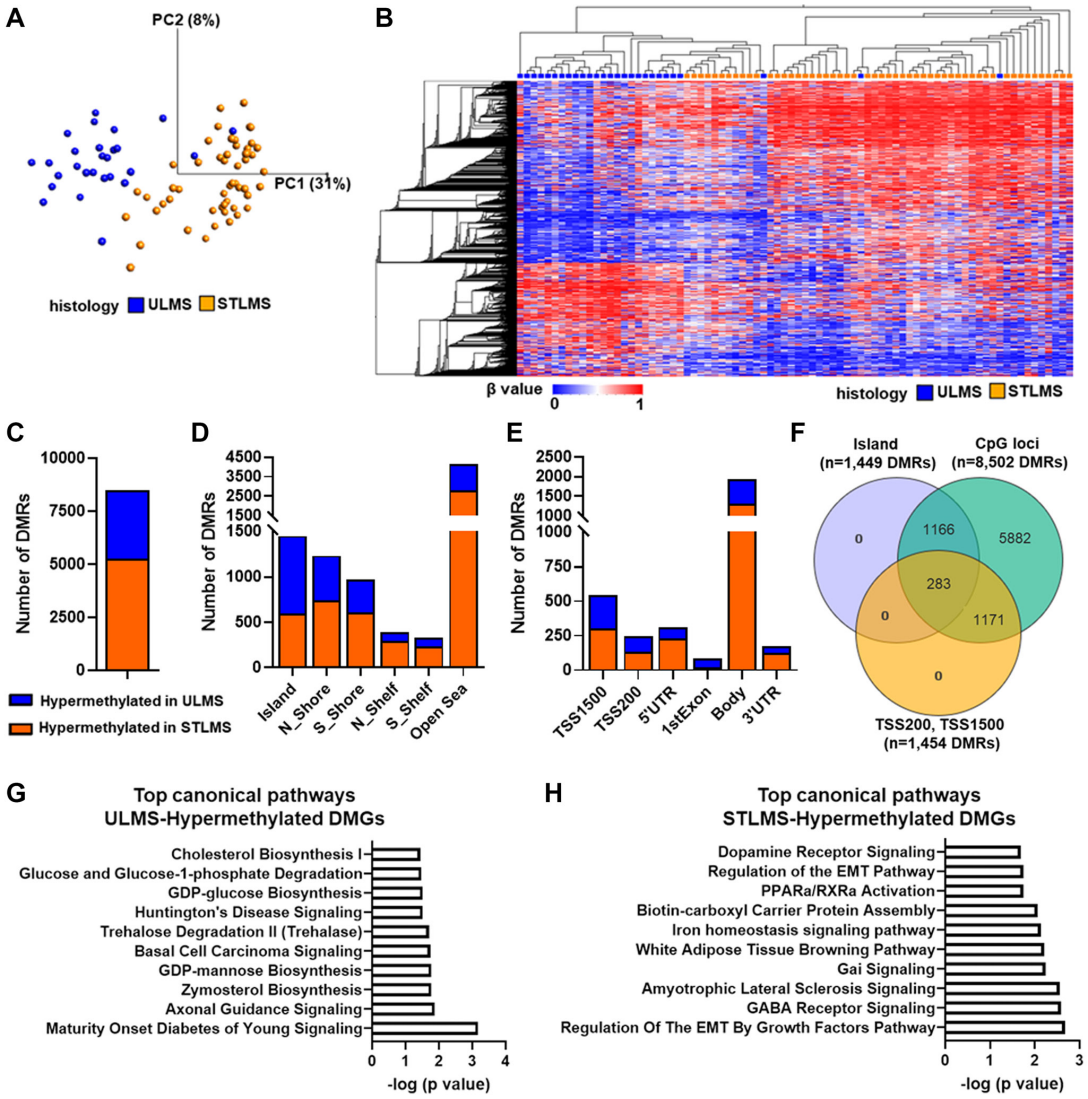
DNA methylation landscape of LMS. (**A**) Principal Component Analysis of STLMS (orange) and ULMS (blue) samples based on DMRs (*n* = 8,502). x- and y-axis show the principal components (PCs). Numbers in brackets indicate the percentage variance for each PC. (**B**) Unsupervised hierarchical clustering analysis of DMRs in ULMS and STLMS. Samples are presented in columns and the DMRs (*n* = 8,502) are presented in rows. Variables were sorted by hierarchical clustering on both the x-axis and the y-axis. The heatmap scale for methylation is based on β values (ranging from 0 (unmethylated) to 1 (methylated)). (**A**, **B**) Analysis was performed using the following filters in Qlucore: 5% variance, *q*-value < 0.05 (*t*-test) and β difference (Δβ (β^ULMS^ − β^STLMS^) > |0.2|). (**C**) Distribution of DMRs based on methylation status. (**D**) Distribution of DMRs in different genomic regions relative to CpG islands including CpG islands, CpG shores and CpG shelves. (**E**) Distribution of DMRs relative to gene region features (TSS1500, TSS200, 5’UTR, 1st exon, gene body, and 3’UTR). Probes binding to multiple different gene regions and intergenic probes are not included in this graph. Please see Supplementary Table 2 for detailed distribution of the DMRs. (**F**) Venn diagram of the DMRs (*n* = 8,502), DMRs mapped to the island region (*n* = 1,449) and DMRs mapped to TSS200-TSS1500 regions (including probes binding to multiple regions that include TSS 200 and/or TSS 1500) (*n* = 1,454)). (**G**) Top 10 canonical pathways associated with ULMS-hypermethylated DMGs (*n* = 77). (**H**) Top 10 canonical pathways associated with STLMS-hypermethylated DMGs (*n* = 73). (**G**, **H**) Analysis was performed in Ingenuity Pathway Analysis using the following cutoffs: −log (*p*-value) >1.3. A detailed list of the canonical pathways is shown in Supplementary Table 4 and Supplementary Table 6.

Data from LMS primary tumor samples from the TCGA-SARC cohort was used for comparative DNA methylation analysis (Figure 1). After pre-filtering and statistical filtering, 8,502 differentially methylated CpG regions (DMRs) were found to be statistically significant (*q*-value < 0.05, Δβ^ULMS-STLMS^ > |0.2|) between ULMS and STLMS and used for further analysis (Figure 2). Principal Component Analysis (PCA) of the DNA methylation data revealed separation between ULMS and STLMS. The majority of the STLMS and ULMS samples segregated apart and formed spatially distinct clusters (Figure 2A). Heatmap representation of an unsupervised hierarchical clustering analysis (HCA) based on the DMRs demonstrated that the majority of the LMS samples segregate into two main clusters based on the subtype status: ULMS-enriched cluster and STLMS-enriched cluster (Figure 2B).

Next, we classified these DMRs based on their methylation levels. Among the 8,502 DMRs that passed the statistical filtering, 5,239 (51.6%) were hypermethylated in STLMS, and 3,263 (38.4%) were hypermethylated in ULMS (Figure 2C). Heatmap representation of an unsupervised HCA based on the 3,263 DMRs hypermethylated in ULMS demonstrated the distinct separation of ULMS and STLMS samples (Supplementary Figure 1). The DMRs were further analyzed in reference to the CpG island loci. Of the 8,502 DMRs, 1,449 DMRs were located in the Island regions, 717 DMRs in the Shelf regions, and 2,197 DMRs in the Shore regions. Interestingly, ULMS was associated with a higher percentage of hypermethylated DMRs (26.2%) located in the Island regions compared to STLMS (11.3%) (Figure 2D, Supplementary Table 1). Next, we analyzed the distribution profile of the DMRs in reference to the gene regions (Figure 2E, Supplementary Table 2). A higher percentage of DMRs mapping to TSS1500, TSS200, 1st Exon regions were hypermethylated in ULMS, whereas a higher percentage of DMRs mapping to 5’UTR, 3’UTR and Body regions were hypermethylated in STLMS (Figure 2E, Supplementary Table 2). We next focused on the promoter-associated CpG island regions and identified 283 DMRs that were associated with 150 differentially methylated genes (DMGs) (Figure 2F, Supplementary Table 3), of which 77 were hypermethylated in ULMS and 73 were hypermethylated in STLMS.

Ingenuity pathway analysis was used to classify the canonical pathways associated with these DMGs. First, we performed pathway analysis using the 77 ULMS-Hypermethylated DMGs and identified 18 canonical pathways (Figure 2G, Supplementary Table 4), primarily associated with metabolic pathways and signaling, axonal guidance and basal cell carcinoma signaling. Interestingly, the top upstream regulators included chromatin modifying enzymes (KAT6A, KMT2A, EZH2) and chromatin/DNA binding proteins (CTNNB1, PBX3, SATB1, MEIS, COMMD1-BMI1) suggesting the possible involvement of chromatin modulation in regulating the DNA methylation of these DMGs (Supplementary Table 5). Pathway analysis of the 73 STLMS-Hypermethylated DMGs revealed 21 canonical pathways (Figure 2H, Supplementary Table 6) associated with EMT, metabolic pathways, and signaling. The top upstream regulators again included chromatin modifying enzymes (KMT2A, EZH2) and chromatin/DNA binding proteins (MLLT1, HOXA11, LBX1, PHF1, SIX1, GSC) (Supplementary Table 7).

### Characterization of differential transcriptional signatures between ULMS and STLMS

**Figure 3:**
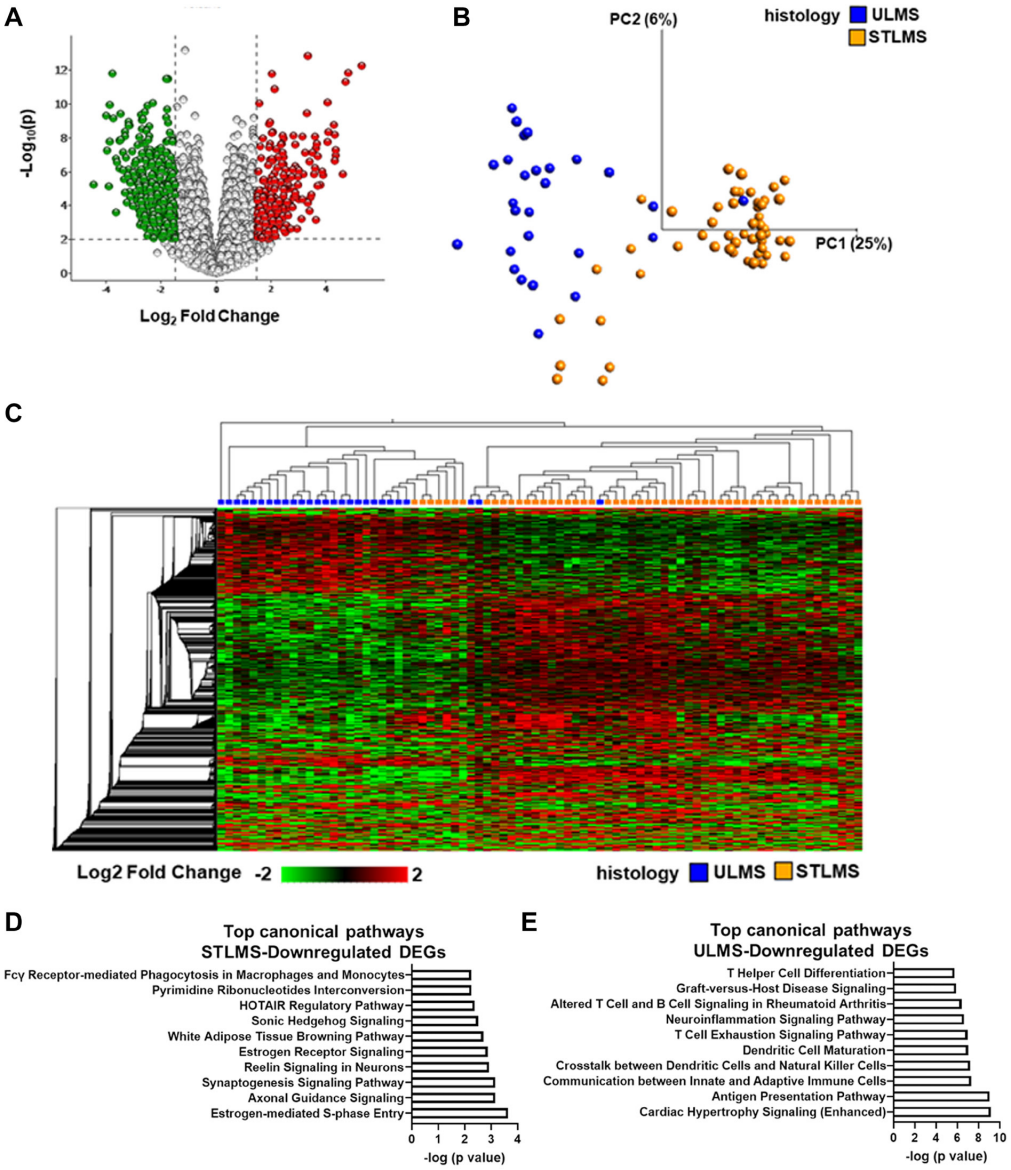
Profiling of differentially expressed genes in LMS. (**A**) Volcano plot of the global transcriptional changes in ULMS relative to STLMS. Each circle represents one gene. Statistical cut-off values are indicated by the grey lines: *p* ≤ 0.01 (corresponding to q-value < 0.05) and log2 fold change (FC) > |1.5|. The colored circles are the DEGs that pass the statistical filtering step (*n* = 2,196) (red-higher expression in ULMS (lower expression in STLMS), green-lower expression in ULMS (higher expression in STLMS)). y-axis indicates the minus log10 of *p*-value for gene, and x-axis shows the log2 FC between ULMS and STLMS. (**B**) Principal Component Analysis of STLMS (orange) and ULMS (blue) samples. x- and y-axis show the PCs. Numbers in brackets indicate the percentage variance for each PC. (**C**) Unsupervised heatmap representing color-coded expression levels of DEGs in ULMS relative to STLMS. Variables were sorted by hierarchical clustering on both the x-axis and the y-axis. The heatmap colors are based on gene expression, with red being upregulated and green being downregulated. Analysis in B–C was performed using the following filters in Qlucore: 5% variance, q-value < 0.05 (*t*-test), log2 FC > |1.5|. (**D**) Top 10 canonical pathways associated with STLMS-downregulated DEGs (*n* = 843). (**E**) Top 10 canonical pathways associated with ULMS-downregulated DEGs (*n* = 1,343). (G, H) Analysis was performed in Ingenuity Pathway Analysis using the following cutoffs: *q*-value < 0.05, log2 FC > |1.5|, −log (*p*-value) > 1.3. A detailed list of the canonical pathways is shown in Supplementary Table 8 and Supplementary Table 9.

Next, we determined the transcriptional differences between ULMS and STLMS by comparing the RNA-seq data of the LMS samples from the TCGA-SARC database. With filtering criteria of *q*-value < 0.05 and log2 fold change (FC) > |1.5|), 2,196 differentially expressed genes (DEGs) were identified (Figure 3A). Similar to the DNA-methylation based clustering, the majority of the STLMS and ULMS samples segregated apart and formed spatially distinct clusters on the PCA plot (Figure 3B). Unsupervised HCA again demonstrated two main clusters on the heatmap: ULMS-enriched cluster and STLMS-enriched cluster (Figure 3C). 1,353 DEGs had lower expression in ULMS, whereas 843 DEGs had lower expression in STLMS. Pathway analysis of the DEGs with lower expression in STLMS revealed 60 canonical pathways (*p* < 0.05), including metabolic pathways, estrogen signaling, neuronal signaling, and development (Figure 3D, Supplementary Table 8). On the other hand, pathway analysis of the DEGs with lower expression in ULMS revealed 199 canonical pathways (*p* < 0.05), the top pathways being associated with neuronal signaling and immune pathways (Figure 3E, Supplementary Table 9).

### Integrated analysis of differentially methylated and expressed genes

**Figure 4:**
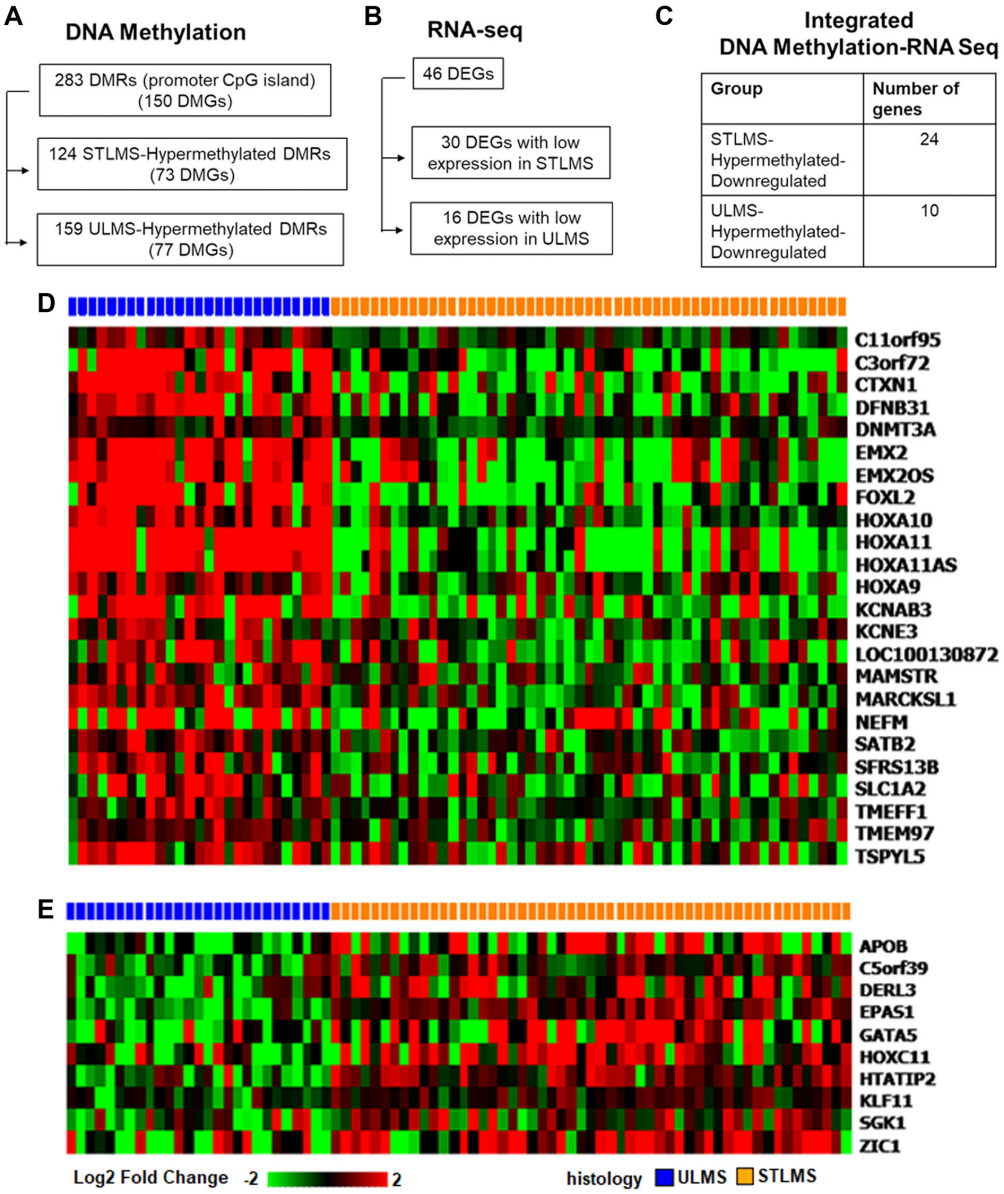
Integrated analysis of differentially methylated and expressed genes in ULMS and STLMS. (**A**) Summary of DMRs mapped to CpG island regions and TSS200/TSS1500 regions (*q*-value < 0.05, Δβ > |0.2|). (**B**) Summary of DEGs corresponding to DMGs in panel A (*q* < 0.05, log2 FC > |1.5|). (**C**) Genes grouped according to the methylation and expression changes in ULMS and STLMS. (**D**) Supervised clustering of hypermethylated and downregulated genes in STLMS. (**E**) Supervised clustering of hypermethylated and downregulated genes in ULMS.

Analysis of the DNA methylation profiles of ULMS and STLMS revealed 283 promoter CpG island-associated DMRs corresponding to 150 DMGs (Figure 4A). Integrated analysis of the DEGs identified 46 genes to be both differentially methylated and differentially expressed (DM-DEGs) (Figure 4B), that were further grouped into two categories: ULMS-Hypermethylated-Downregulated (10 genes) and STLMS-Hypermethylated-Downregulated (24 genes) (Figure 4C). Overall, 34 genes out of the 46 DM-DEGs (73.9%) demonstrated the expected inverse correlation between promoter CpG island methylation and gene expression. The supervised analysis demonstrated that these DM-DEGs can distinguish between ULMS and STLMS (Figure 4D, Figure 4E). The remaining 12 out of the 46 genes had a positive correlation between DNA methylation and gene expression, suggesting that the regulation of gene expression is a complicated process and can involve other epigenetic mechanisms, such as chromatin structure, and non-epigenetic mechanisms.

**Figure 5:**
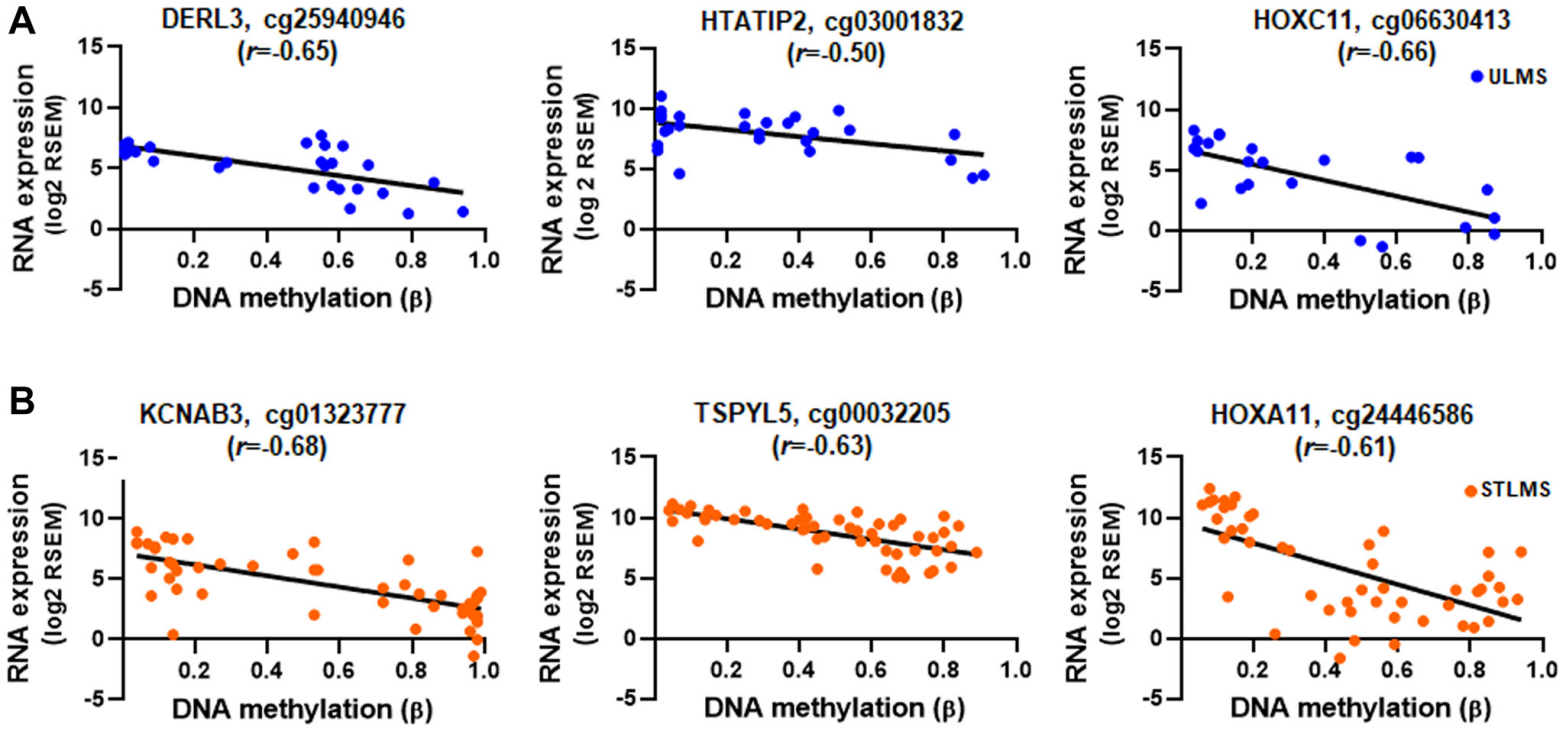
Correlation between gene expression and DNA methylation. RNA expression and DNA methylation values for the genes with moderate correlation ((|0.5| < r < |0.7|), *p* < 0.01) for ULMS-Hypermethylated-Downregulated (**A**) and STLMS-Hypermethylated-Downregulated group (**B**). Shown are the top 3 genes in each group. RNA expression values are plotted as log2 RSEM and DNA methylation are plotted as β values. Detailed analysis results are shown in Supplementary Table 10.

Next, we assessed the correlation between DNA methylation and gene expression in the ULMS-Hypermethylated-Downregulated and STLMS-Hypermethylated-Downregulated groups by determining the Pearson correlation coefficient (*r*). Moderate correlation (|0.5| < *r* < |0.7|) was found for genes in both ULMS (DERL3, HOXC11, HTATIP2, and C5orf39) and STLMS (KCNAB3, KCNE3, TSPYL5, HOXA11, HOXA11AS, HOX9, LOC100130872) (Figure 5A, Figure 5B, Supplementary Table 10).

**Figure 6:**
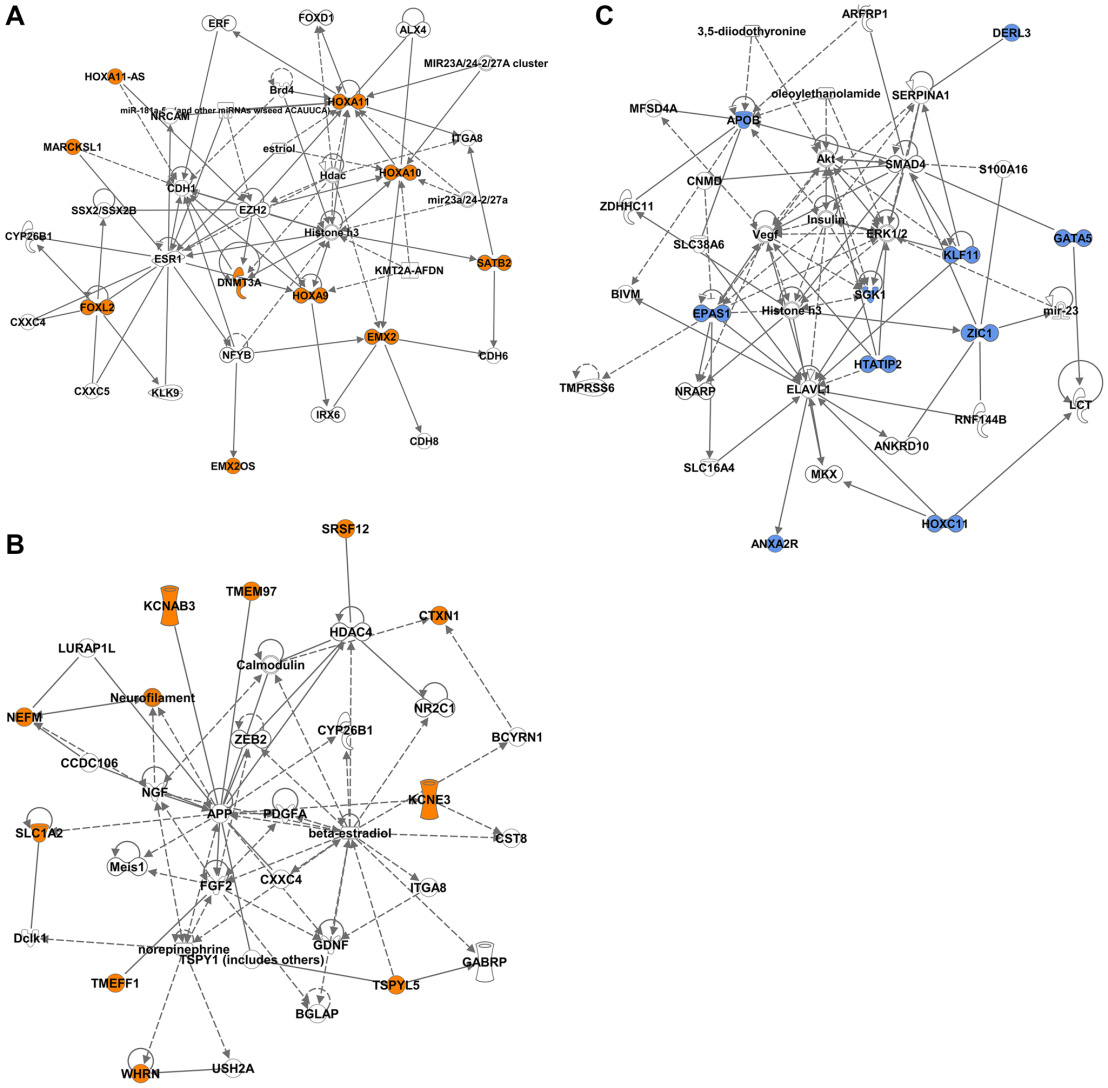
Network analysis of the DM-DEGs associated with STLMS-Hypermethylated-Downregulated and ULMS-Hypermethylated-Downregulated groups. Ingenuity network analysis was used to plot the gene relationships. The colored shapes indicate the downregulated genes in the STLMS-Hypermethylated-Downregulated group (orange color) (**A**, **B**) and the ULMS-Hypermethylated-Downregulated groups (blue color) (**C**). Genes that do not have corresponding colors, were not identified as differentially expressed in our analysis, and were integrated based on the Ingenuity Pathway Analysis evidence indicating a relevance to this network.

Interaction network analysis of the DM-DEGs revealed two networks associated with the STLMS-Hypermethylated-Downregulated group and one network associated with the ULMS-Hypermethylated-Downregulated group (Figure 6). Potential interacting proteins that emerged in the STLMS-Hypermethylated-Downregulated group again included proteins related to epigenetic regulation (EZH2, Histone H3, KMT2A-AFDN, BRD4, HDAC, HDAC4), neurofilament/neuronal signaling (NEFM, NGF, APP), growth factors (NGF, FGF2, PDGFA, GDNF), metastasis (ZEB2), and hormonal receptors (ESR1) (Figure 6A, Figure 6B). Potential interacting proteins in the ULMS-Hypermethylated-Downregulated group included genes related to transcriptional regulation (SMAD4), signaling (Akt, ERK1/2), and epigenetic regulation (Histone H3) (Figure 6C).

## DISCUSSION

LMS is associated with a poor prognosis due to its aggressive nature, heterogeneous origin, and high risk for recurrence and metastasis. LMS may arise from uterine tissues or a wide array of soft tissues and has a variable prognosis. However, limited research has been done to understand if these are distinct tumors. A previous study by the TCGA across all soft tissue sarcoma subtypes showed that soft tissue sarcomas have a low somatic mutation burden and predominant copy number alterations [18]. Unsupervised analysis of the TCGA-SARC data using the iCluster approach clustered soft tissue sarcomas to five clusters. STLMS and ULMS clustered together as a spatially distinct cluster compared to other sarcomas, showing that ULMS and STLMS shared similar profiles. The analysis also showed that ULMS and STLMS shared different methylation and mRNA expression profiles.

Studies on the epigenetic regulation of LMS have been limited so far [18, 20, 21, 30, 31]. Most of the studies have focused on the characterization of epigenetic profiles of all sarcoma subtypes. Analysis of DNA methylation data from different sarcoma subtypes and other tumors revealed that LMS samples clustered separately and suggested that DNA methylation data could be used as a diagnostic tool [20, 21, 31]. A previous study by our group also revealed that LMS has exquisite sensitivity to epigenetic drugs such as hypomethylating agents [28]. Thus, epigenetic profiling of LMS can lead to the identification of novel drug targets and biomarkers as shown by multiple studies by our group and others in different cancer types [32, 33].

In this study, we performed a comprehensive analysis and compared the DNA methylation and RNA expression profiles of ULMS and STLMS samples from the TCGA-SARC study. Unsupervised hierarchical clustering of DMRs showed the presence of two main clusters: ULMS-enriched cluster and STLMS-enriched cluster. The ULMS-enriched cluster contained a subset of the STLMS samples (11 out of 53, 20.7%). This data is in agreement with the previous study showing the presence of two clusters of STLMS with different methylation profiles [18]. Further analysis identified 283 DMRs in the promoter-associated CpG island sites that differ between ULMS and STLMS. Upstream regulator analysis of the DMGs through Ingenuity Pathway Analysis revealed chromatin-modifying enzymes, DNA-binding and chromatin-binding proteins suggesting the possible involvement of chromatin modulation in the oncogenesis of LMS subtypes. Studies in other solid tumors also pointed to the role of chromatin dysregulation in cancer formation [34]. Majority of the canonical pathways associated with the DMGs pointed to metabolic pathways, neuronal pathways, and EMT signaling (Figure 2). Furthermore, methylation of promoter-associated CpG islands has been shown to downregulate expression and we identified 34 DM-DEGs with the expected negative correlation between promoter CpG island methylation and gene expression (Figure 4, Table 1). Of interest, majority of these genes are transcriptional regulators (13 out of 34, 38.2%), strongly suggestive of different tumor biology driving the subtypes of LMS, and likely different therapeutic targets. In addition, we also noticed that one of the genes is a DNA methyltransferase enzyme suggesting the potential epigenetic regulation of LMS subtypes. Network analysis of the DM-DEGs also pointed to their potential interaction with proteins involved in epigenetic regulation (Figure 6). Further studies will be needed to study the possible roles of these proteins as potential biomarkers or targets for epigenetic drugs.

**Table 1:** List of the genes associated with differential DNA methylation and gene expression in ULMS and STLMS

**Gene name**	**Gene expression** **(log2 FC relative to STLMS)**	**DNA methylation** **(Δβ (β^ULMS^- β^STLMS^) > 0.2)**	**Entrez Gene Name**	**Location**	**Type(s)**
APOB	0.23	0.34	apolipoprotein B	Extracellular Space	transporter
DERL3	0.27	0.29	derlin 3	Cytoplasm	other
EPAS1	0.30	0.21	endothelial PAS domain protein 1	Nucleus	transcription regulator
HOXC11	0.23	0.22	homeobox C11	Nucleus	transcription regulator
HTATIP2	0.32	0.24	HIV-1 Tat interactive protein 2	Nucleus	transcription regulator
KLF11	0.61	0.31	Kruppel like factor 11	Nucleus	transcription regulator
SGK1	0.56	0.25	serum/glucocorticoid regulated kinase 1	Cytoplasm	kinase
ZIC1	0.11	0.26	Zic family member 1	Nucleus	transcription regulator
C5orf39	0.50	0.29	Annexin A2 receptor (ANXA2R)	Plasma Membrane	other
GATA5	0.17	0.27	GATA binding protein 5	Nucleus	transcription regulator
**Gene name**	**Gene expression** **(log2 FC relative to ULMS)**	**DNA methylation** **(Δβ (β^STLMS^- β^ULMS^) > 0.2)**	**Entrez Gene Name**	**Location**	**Type(s)**
C11orf95	0.54	0.22	chromosome 11 open reading frame 95	Other	other
C3orf72	0.07	0.23	FOXL2 neighbor (FOXL2NB)	Other	other
CTXN1	0.14	0.26	cortexin 1	Other	other
DFNB31	0.18	0.23	whirlin	Plasma Membrane	other
DNMT3A	0.61	0.21	DNA methyltransferase 3 alpha	Nucleus	enzyme
EMX2*	0.08	0.25	empty spiracles homeobox 2	Nucleus	transcription regulator
EMX2OS*	0.08	0.25	EMX2 opposite strand/antisense RNA	Other	other
HOXA10	0.24	0.30	homeobox A10	Nucleus	transcription regulator
HOXA11*	0.03	0.30	homeobox A11	Nucleus	transcription regulator
HOXA11AS*	0.04	0.30	HOXA11 antisense RNA	Other	other
HOXA9	0.44	0.27	homeobox A9	Nucleus	transcription regulator
KCNAB3	0.13	0.32	potassium voltage-gated channel subfamily A regulatory beta subunit 3	Plasma Membrane	ion channel
KCNE3	0.56	0.23	potassium voltage-gated channel subfamily E regulatory subunit 3	Plasma Membrane	ion channel
LOC100130872	0.28	0.38	uncharacterized	other	other
MAMSTR	0.52	0.20	MEF2 activating motif and SAP domain containing transcriptional regulator	Nucleus	transcription regulator
MARCKSL1	0.32	0.22	MARCKS like 1	Cytoplasm	other
NEFM	0.15	0.24	neurofilament medium	Plasma Membrane	other
SATB2	0.44	0.23	SATB homeobox 2	Nucleus	transcription regulator
SFRS13B	0.43	0.22	serine and arginine rich splicing factor 12 (SFRS12)	Nucleus	other
SLC1A2	0.36	0.29	solute carrier family 1 member 2	Plasma Membrane	transporter
TMEFF1	0.58	0.22	transmembrane protein with EGF like and two follistatin like domains 1	Plasma Membrane	other
TMEM97	0.61	0.24	transmembrane protein 97	Extracellular Space	other
FOXL2	0.06	0.23	forkhead box L2	Nucleus	transcription regulator
TSPYL5	0.42	0.22	TSPY like 5	other	other

Listed are genes in the ULMS-Hypermethylated-Downregulated and STLMS-Hypermethylated-Downregulated groups. For genes associated with multiple probes, the average value per gene was presented. Details about the *q*-values and DNA methylation probes are in Supplementary Table 3 (^*^same probes).

12 DM-DEGs were associated with a positive correlation between methylation of promoter-associated CpG islands and gene expression. 6 DM-DEGs were hypomethylated and upregulated in STLMS and 6 DM-DEGs were hypomethylated and upregulated in ULMS, suggesting that the expression of these genes might be regulated through other mechanisms. Other studies have also pointed to a positive association of DNA methylation and gene expression in cancer [35, 36], suggesting the diversity in epigenetic regulation.

The canonical pathway analysis of DMGs revealed multiple metabolic pathways (Figure 2G, Figure 2H). Numerous studies have pointed to the importance of tumor metabolism and treatment response [37]. Variety of drugs targeting the tumor metabolism are already available [38] and can potentially be combined with immunotherapy agents to facilitate metabolic reprogramming and enhance the antitumor response.

A possible explanation for the epigenetic differences is the tissue sites from which the tumors were derived. Comprehensive studies comparing normal tissue and tumor tissue are needed to further delineate the cancer-specific epigenetic changes at each tissue site. A recent study focused on the DNA methylation-based profiling of sarcomas and included samples corresponding to control tissue and STLMS [20]. We compared these two groups and identified 92 promoter CpG island-associated DMRs that were shared with the DMRs identified in the ULMS-STLMS comparison (Supplementary Table 11). 56 of these DMRs were associated with hypermethylation in STLMS compared to control and hypermethylation in STLMS compared to ULMS. Another study utilizing a small number of samples has demonstrated the differences in DNA methylation and gene expression between normal myometrium and ULMS [30]. We compared these two groups and identified 15 promoter CpG island-associated DMRs common with the DMRs identified in the ULMS-STLMS comparison (Supplementary Table 12). 14 of these DMRs were associated with hypomethylation in ULMS compared to STLMS and hypomethylation in ULMS compared to control. Although the number of common DMRs is small, these comparisons pointed to possible epigenetic regulation through hypermethylation or hypomethylation of promoter CpG island-associated DMRs in ULMS and STLMS compared to control tissues. Similar studies need to be performed with a larger number of LMS and control tissue samples to delineate the LMS-specific molecular changes independent of the site of origin.

In summary, we show evidence for differential DNA methylation profiles between ULMS and STLMS suggesting that differential epigenetic profiles are associated with LMS subtypes and may be responsible for differences seen in clinical outcomes. These findings suggest that epigenetic profiles can be used to stratify patients and apply therapeutic agents targeting the epigenetic mechanisms for LMS management. We identified several DM-DEGs and associated pathways. These findings can be used to improve our understanding of epigenetic regulation and clinical outcomes in LMS subtypes and guide biomarker development or targeted therapies.

## MATERIALS AND METHODS

### Datasets used in the study

We used data corresponding to the LMS cases from the TCGA-SARC data. Both the DNA methylation data (Illumina Infinium HumanMethylation450 BeadChip, level 3) and normalized expression data (RNA-seqV2, level 3) for ULMS and STLMS cases (*n* = 80 samples, 27 ULMS, 53 STLMS) were downloaded from the Broad Institute GDAC FireBrowse portal. The analysis focused on primary tumor samples and the tumor subtype classification was defined by the TCGA clinical data [18]. The tumor sites for ULMS included uterus (*n* = 24) and abdomen (*n* = 3), whereas the tumor sites for STLMS included abdomen, primarily retroperitoneum (*n* = 36), extremities (14) and other regions (*n* = 3).

Additional public datasets were interrogated for DNA methylation including GSE140686 (*n* = 24 samples; 8 controls, 16 STLMS; Illumina HumanMethylation450 BeadChips or Illumina Infinium HumanMethylation850 BeadChips) [20] and GSE68312 (*n* = 5 samples; 3 controls, 2 ULMS Stage I; Illumina Infinium HumanMethylation450 BeadChip [30] and were downloaded from the Gene Expression Omnibus (http://www.ncbi.nlm.nih.gov/geo/).

### Analysis of DNA methylation data

DNA methylation data for the TCGA-SARC dataset was analyzed as follows. First, the following CpG sites were removed from the analysis: 1. CpG sites with missing values >80% across the LMS cohort, 2. CpG sites on the X and Y chromosomes (potential bias due to female gender association with ULMS), 3. CpG sites on single nucleotide polymorphisms (SNPs), and 4. Cross-reactive probes [39]. A final set of 249,163 CpG sites was further analyzed. DNA methylation levels of CpG sites were calculated as β values. Probes were subjected to analysis with Qlucore Omics Explorer (v 3.6). Missing values were reconstructed using the average function. For stringent data filtering and visualization, data was first sorted by a variance of 5%. The average β values of the STLMS samples and the ULMS samples were calculated. Statistical filtering (*q*-value < 0.05 and Δβ^ULMS-STLMS^ > |0.2|) was applied to identify the differentially methylated regions (DMRs) between ULMS and STLMS groups. The DMRs were divided into two main groups: ULMS-hypermethylated (Δβ^ULMS-STLMS^ > 0.2) and STLMS-hypermethylated (Δβ^STLMS-ULMS^ > 0.2) and classified into subcategories depending on the 1. Location relative to the CpG island (CpG island, N_Shore, S_Shore, N_Shelf, S_Shelf, and Open Sea) and 2. Location based on the gene features (TSS1500, TSS200, 5’UTR, 1st Exon, Gene Body, 3’UTR, intergenic region). CpG site and gene mapping files were downloaded from https://www.illumina.com/. Genes with DMRs were described as differentially methylated genes (DMGs).

DNA methylation data for control muscle samples (reference samples 1-8) and STLMS samples (reference samples 116–120, 185, 186, 261, 391, 397, 680–683, 931, 1050) were extracted from the GSE140686 dataset [20]. Variables were analyzed with Qlucore Omics Explorer (v 3.6) using the following settings: variance = 5%, *q*-value < 0.05 and Δβ^STLMS-CONTROL^ > |0.2|) to identify the DMRs in STLMS compared to control groups.

DNA methylation data for control normal myometrium (samples 1-3) and ULMS (stage I, samples 2–3) were extracted from the GSE68312 dataset [30]. Variables were analyzed with Qlucore Omics Explorer (v 3.6) using the following settings: variance = 5%, *q*-value < 0.05 and Δβ^ULMS-CONTROL^ > |0.2|) to identify the differentially methylated regions (DMRs) between ULMS and control groups.

### Analysis of gene expression data

Transcripts with missing values >80% across the LMS cohort were removed. A final set of 16,179 transcripts was further analyzed. Differential gene expression was determined using Qlucore Omics Explorer (v 3.6). A threshold of 0.01 was used and missing values were reconstructed using the average function. Log2 transformed RSEM values were used for all calculations and data representation. For stringent data filtering and visualization, data was first sorted by variance of 5%. Then, statistical filtering using *q*-value < 0.05 and log2 fold change > |1.5| was applied to determine the differentially expressed genes (DEGs) between ULMS and STLMS. Fold change was calculated as 2 to the power of the average difference between the two groups.

### Integrated analysis of DNA methylation and gene expression

Normalized gene expression and DNA methylation array data were integrated based on the common genes identified as DEGs in the gene expression analysis and DMGs in the DNA methylation array. For this integrated analysis only DMGs with DMRs in CpG island regions upstream of TSS (within 1500 bp) were included. To study the relationship between gene methylation and expression, DM-DEGs (the genes in the intersection of DMGs and DEGs) were classified into two distinct groups: ULMS-Hypermethylated-Downregulated and STLMS-Hypermethylated- Downregulated.

### Ingenuity pathway analysis for DNA methylation data

DNA methylation difference and *q*-value data comparing ULMS and STLMS was derived from Qlucore Omics Explorer (v 3.6). The DMGs corresponding to DMRs associated with promoter CpG islands (*q*-value < 0.05, Δβ^ULMS-STLMS^ > |0.2|) were uploaded to Ingenuity Pathway Analysis (Qiagen) for canonical pathway and upstream regulator analysis. Data for 77 ULMS-Hypermethylated DMGs and 73 STLMS-Hypermethylated DMGs were used. Canonical pathways with a *p*-value < 0.05 (-log (*p*-value) < 1.3) were considered as statistically significant.

### Ingenuity pathway analysis for RNA-seq data

Log2 fold change and *q*-value data comparing ULMS and STLMS was derived from Qlucore Omics Explorer (v 3.6) and uploaded to Ingenuity Pathway Analysis (Qiagen)for pathway analysis. DEGs were filtered based on *q*-value < 0.05 and log2 fold change > |1.5| and used for further analysis. Canonical pathways with a *p*-value < 0.05 (or -log (*p*-value) < 1.3) were considered statistically significant.

## SUPPLEMENTARY MATERIALS





## Abbreviations

LMS: Leiomyosarcoma
ULMS: Uterine Leiomyosarcoma
STLMS: Soft Tissue Leiomyosarcoma
TCGA: The Cancer Genome Atlas
PCA: Principal Component Analysis
HCA: Hierarchical Clustering Analysis
FC: Fold Change
DMRs: Differentially Methylated Regions
DMGs: Differentially Methylated Genes
DEGs: Differentially Expressed Genes
