# Supplementary material for: Epigenetic signatures differentiate uterine and soft tissue leiomyosarcoma
Source: Oncotarget. 2021 Aug 3;12(16):1566–79. doi: 10.18632/oncotarget.28032 (PMC8351604; doi:10.18632/oncotarget.28032)
Supplement: Supplementary file 1 [file oncotarget-12-1566-s001.pdf]

Epigenetic signatures differentiate uterine and soft tissue leiomyosarcoma

SUPPLEMENTARY MATERIALS

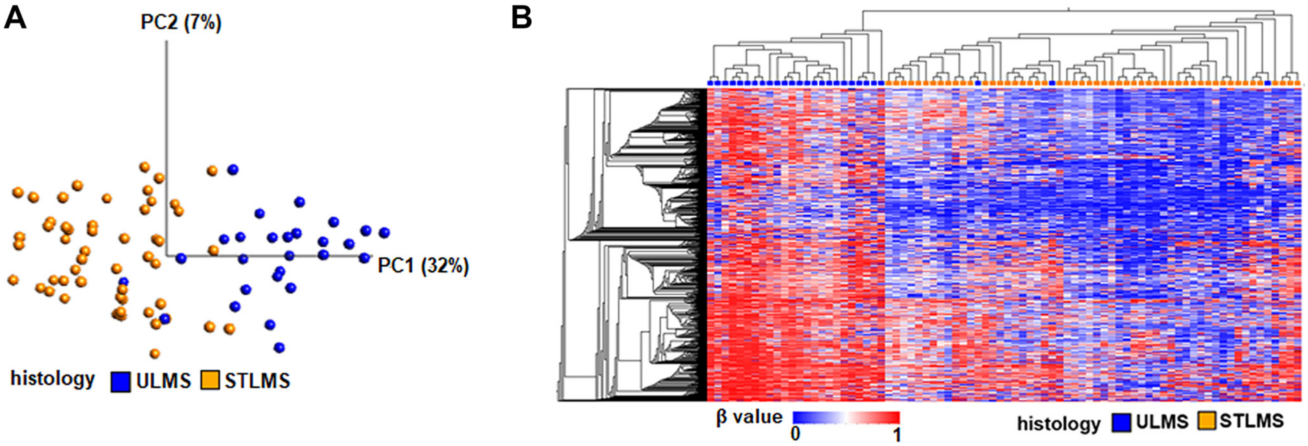

Supplementary Figure 1: DNA methylation landscape of DMRs hypermethylated in ULMS. (**A**) Principal Component Analysis of STLMS (orange) and ULMS (blue) samples based on DMRs hypermethylated in ULMS (n = 3,263). (**B**) Unsupervised hierarchical clustering analysis of DMRs hypermethylated in ULMS relative to STLMS. Samples are presented in columns and the DMRs (n = 3,263) are presented in rows. The heatmap scale for methylation is β values (ranging from 0 (unmethylated) to 1 (methylated). (A, B) Analysis was performed using the following filters in Qlucore: 5% variance, q-value < 0.05 (t-test) and β difference (Δβ ($\beta^{ULMS} - \beta^{STLMS}$) > |0.2|).

Supplementary Table 1: Classification of DMRs based on methylation status and CpG island loci distribution

	Island	N_Shore	N_Shelf	S_Shore	S_Shelf	Open Sea	Total
**Hypermethylated in ULMS**	855 (26.2%)	491 (15.0%)	94 (2.9%)	361 (11.1%)	98 (3.0%)	1364 (41.8)	3263
**Hypermethylated in STLMS**	594 (11.3%)	740 (14.1%)	291 (5.6%)	605 (11.5%)	234 (4.5%)	2775 (53.0%)	5239

Supplementary Table 2: Classification of DMRs based on methylation status and gene regions

	TSS1500	TSS200	5′UTR	1stExon	3′UTR	Body	Multiple	Intergenic	Total
**Hypermethylated in ULMS**	246 (7.5%)	112 (3.4%)	86 (2.6%)	66 (2.0%)	49 (1.5%)	629 (19.3%)	714 (21.9%)	1361 (41.7%)	3263
**Hypermethylated in STLMS**	302 (5.8%)	132 (2.5%)	227 (4.3%)	21 (0.4%)	124 (2.4%)	1298 (24.8%)	1614 (30.8%)	1521 (29.0%)	5239

Supplementary Table 3: Details of the 283 DMRs located at CpG island loci and mapped to TSS 200 and/or TSS 1500 regions. See Supplementary Table 3

Supplementary Table 4: Canonical pathways associated with ULMS-hypermethylated DMGs

	Ingenuity Canonical Pathways	−log (p-value)	Ratio	z-score	Genes
1	Maturity Onset Diabetes of Young (MODY) Signaling	3.17	0.05	NaN	APOB, KLF11,ONECUT1
2	Axonal Guidance Signaling	1.88	0.01	NaN	GDF7, GNG4, HHIP, NGEF, WNT6
3	Zymosterol Biosynthesis	1.77	0.17	NaN	CYP51A1
4	GDP-mannose Biosynthesis	1.77	0.17	NaN	GMPPA
5	Basal Cell Carcinoma Signaling	1.74	0.03	NaN	HHIP, WNT6
6	Trehalose Degradation II (Trehalase)	1.7	0.14	NaN	HK1

**7**	Huntington's Disease Signaling	1.51	0.01	NaN	GNG4, SGK1, STX16
**8**	GDP-glucose Biosynthesis	1.51	0.09	NaN	HK1
**9**	Glucose and Glucose-1-phosphate Degradation	1.47	0.08	NaN	HK1
**10**	Cholesterol Biosynthesis I	1.44	0.08	NaN	CYP51A1
**11**	NAD Phosphorylation and Dephosphorylation	1.44	0.08	NaN	ACP5
**12**	Cholesterol Biosynthesis II (via 24, 25-dihydrolanosterol)	1.44	0.08	NaN	CYP51A1
**13**	Cholesterol Biosynthesis III (via Desmosterol)	1.44	0.08	NaN	CYP51A1
**14**	UDP-N-acetyl-D-galactosamine Biosynthesis II	1.44	0.08	NaN	HK1
**15**	Colanic Acid Building Blocks Biosynthesis	1.41	0.07	NaN	GMPPA
**16**	Role of NANOG in Mammalian Embryonic Stem Cell Pluripotency	1.34	0.02	NaN	TCL1A, WNT6
**17**	LXR/RXR Activation	1.33	0.02	NaN	APOB, CYP51A1
**18**	GP6 Signaling Pathway	1.31	0.02	NaN	COL2A1, COL9A1

Supplementary Table 5: Upstream regulators for ULMS-hypermethylated DMGs. See Supplementary Table 5

Supplementary Table 6: Canonical pathways associated with STLMS-hypermethylated DMGs

	Ingenuity Canonical Pathways	−log (*p*-value)	Ratio	z-score	Genes
**1**	Regulation Of The Epithelial Mesenchymal Transition By Growth Factors Pathway	2.67	0.02	NaN	FOXC2, PDGFA, SMAD3, ZEB1
**2**	GABA Receptor Signaling	2.58	0.03	NaN	ADCY1, AP2A2, GABRA2
**3**	Amyotrophic Lateral Sclerosis Signaling	2.55	0.03	NaN	GDNF, NEFM, SLC1A2
**4**	Gαi Signaling	2.24	0.02	NaN	ADCY1, DRD4, RALGDS
**5**	White Adipose Tissue Browning Pathway	2.21	0.02	NaN	ADCY1, FOXC2, PRDM16
**6**	Iron homeostasis signaling pathway	2.13	0.02	NaN	HIF3A, PDGFA, SMAD3
**7**	Biotin-carboxyl Carrier Protein Assembly	2.06	0.33	NaN	HLCS
**8**	PPARα/RXRα Activation	1.74	0.02	NaN	ADCY1, AP2A2, SMAD3
**9**	Regulation of the Epithelial-Mesenchymal Transition Pathway	1.74	0.02	NaN	FOXC2, SMAD3, ZEB1
**10**	Dopamine Receptor Signaling	1.68	0.03	NaN	ADCY1, DRD4
**11**	VDR/RXR Activation	1.67	0.03	NaN	HOXA10, PDGFA
**12**	PEDF Signaling	1.63	0.02	NaN	GDNF, ZEB1
**13**	HIPPO signaling	1.6	0.02	NaN	SMAD3, TEAD3
**14**	Opioid Signaling Pathway	1.46	0.01	NaN	ADCY1, AP2A2, PENK
**15**	Sumoylation Pathway	1.44	0.02	NaN	DNMT3A, ZEB1
**16**	Colorectal Cancer Metastasis Signaling	1.43	0.01	NaN	ADCY1, RALGDS, SMAD3
**17**	Pancreatic Adenocarcinoma Signaling	1.4	0.02	NaN	RALGDS, SMAD3
**18**	Apelin Endothelial Signaling Pathway	1.36	0.02	NaN	ADCY1, SMAD3
**19**	Sphingosine-1-phosphate Signaling	1.34	0.02	NaN	ADCY1, PDGFA
**20**	Chondroitin Sulfate Degradation (Metazoa)	1.34	0.06	NaN	HYAL2
**21**	Dermatan Sulfate Degradation (Metazoa)	1.32	0.06	NaN	HYAL2

Supplementary Table 7: Upstream regulators for STLMS-hypermethylated DMGs. See Supplementary Table 7

Supplementary Table 8: Canonical pathways associated with STLMS-downregulated DEGs. See Supplementary Table 8

Supplementary Table 9: Canonical pathways associated with ULMS-downregulated DEGs. See Supplementary Table 9

Supplementary Table 10: DNA methylation-gene expression correlation

	Pearson correlation coefficient (r)- ULMS samples	R2	*p*-value	correlation
**APOB (cg16723488)**	−0.03	0.000098	0.910	very weak
**DERL3 (probe 1, cg25940946)**	−0.65	0.42	0.000	moderate
**DERL3 (probe 2, cg14774086)**	−0.66	0.44	0.000	moderate
**DERL3 (probe 3, cg25037461)**	−0.64	0.42	0.000	moderate
**EPAS1 (cg21276379)**	−0.26	0.07	0.198	very weak
**HOXC11 (cg06630413)**	−0.66	0.43	0.001	moderate
**HTATIP2 (probe 1, cg03001832)**	−0.5	0.25	0.009	moderate
**HTATIP2 (probe2, cg20344434)**	−0.5	0.25	0.009	moderate
**KLF11 (probe 1, cg26276650)**	−0.08	0.006	0.690	very weak
**KLF11 (probe 2, cg02983451)**	−0.22	0.049	0.270	very weak
**SGK1 (cg08239804)**	0.09	0.008	0.660	very weak
**ZIC1 (probe1, cg01227537)**	−0.39	0.15	0.080	weak
**ZIC1 (probe2, cg14456683)**	−0.29	0.08	0.200	very weak
**C5orf39 (probe 1, cg22055815)**	−0.49	0.24	0.010	weak
**C5orf39 (probe 2, cg03723510)**	−0.46	0.22	0.015	weak
**C5orf39 (probe 3, cg01313313)**	−0.59	0.35	0.001	moderate
**C5orf39 (probe 4, cg05551979)**	−0.46	0.21	0.020	weak
**C5orf39 (probe 5, cg00257271)**	−0.67	0.44	0.000	moderate
**C5orf39 (probe 6, cg17269733)**	−0.53	0.28	0.004	moderate
**GATA5 (probe 1, cg09339194)**	0.07	0.04	0.790	very weak
**GATA5 (probe 2, cg23770904)**	0.16	0.024	0.520	very weak
**GATA5 (probe 3, cg14388488)**	0.3	0.09	0.210	very weak

	Pearson correlation coefficient (r)- STLMS samples	R2	*p*-value	correlation
**C11orf95 (cg04986616)**	−0.46	0.21	0.00	weak
**C3orf72 (cg01379240)**	−0.14	0.02	0.42	very weak
**CTXN1 (probe1, cg10709671)**	−0.19	0.036	0.17	very weak
**CTXN1 (probe 2, cg19458602)**	−0.009	8.17E−05	0.95	very weak
**DFNB31 (cg12877860)**	−0.44	0.198	0.00	weak
**DNMT3A (cg11354105)**	0.015	0.0002	0.91	very weak
**EMX2 (probe 1, cg20348858)***	−0.23	0.05	0.12	very weak
**EMX2 (probe 2, cg16656895)***	−0.29	0.085	0.04	very weak
**EMX2OS (probe 1, cg20348858)***	−0.38	0.14	0.06	weak
**EMX2OS (probe 2, cg16656895)***	−0.41	0.17	0.00	weak
**HOXA10 (probe 1, cg14649140)**	−0.12	0.01	0.40	very weak
**HOXA10 (probe 2, cg09411999)**	−0.15	0.02	0.30	very weak
**HOXA11 (probe 1, cg24709033)***	−0.48	0.24	0.00	weak
**HOXA11 (probe 2, cg09495769)***	−0.4	0.16	0.00	weak
**HOXA11 (probe 3, cg25901381)***	−0.47	0.22	0.00	weak
**HOXA11 (probe 4, cg09661370)***	−0.53	0.28	0.00	moderate
**HOXA11 (probe 5, cg10767141)***	−0.47	0.22	0.00	weak
**HOXA11 (probe 6, cg15760840)***	−0.44	0.2	0.00	weak
**HOXA11 (probe 7, cg10657141)***	−0.57	0.33	<0.0001	moderate
**HOXA11 (probe 8, cg15431544)***	−0.57	0.33	<0.0001	moderate
**HOXA11 (probe 9, cg12810084)***	−0.49	0.24	0.00	weak
**HOXA11 (probe 10, cg05977669)***	−0.63	0.4	<0.0001	moderate
**HOXA11 (probe 11, cg24446586)***	−0.61	0.37	<0.0001	moderate
**HOXA11 (probe 12, cg12997720)***	−0.6	0.36	<0.0001	moderate
**HOXA11AS (probe 1, cg24709033)***	−0.48	0.23	0.00	weak
**HOXA11AS (probe 2, cg09495769)***	−0.49	0.24	0.00	weak

**HOXA11AS (probe 3, cg25901381)***	−0.48	0.23	0.00	weak
**HOXA11AS (probe 4, cg09661370)***	−0.46	0.21	0.00	weak
**HOXA11AS (probe 5, cg10767141)***	−0.48	0.23	0.00	weak
**HOXA11AS (probe 6, cg15760840)***	−0.45	0.2	0.00	weak
**HOXA11AS (probe 7, cg10657141)***	−0.56	0.31	<0.0001	moderate
**HOXA11AS (probe 8, cg15431544)***	−0.62	0.39	<0.0001	moderate
**HOXA11AS (probe 9, cg12810084)***	−0.5	0.25	0.00	moderate
**HOXA11AS (probe 10, cg05977669)***	−0.66	0.44	<0.0001	moderate
**HOXA11AS (probe 11, cg24446586)***	−0.6	0.36	<0.0001	moderate
**HOXA11AS (probe 12, cg12997720)***	−0.62	0.39	<0.0001	moderate
**HOXA9 (cg21942490)**	−0.54	0.3	<0.0001	moderate
**KCNAB3 (probe 1, cg23365801)**	−0.62	0.38	<0.0001	moderate
**KCNAB3 (probe 2, cg13377102)**	−0.63	0.39	<0.0001	moderate
**KCNAB3 (probe 3, cg16513459)**	−0.63	0.39	<0.0001	moderate
**KCNAB3 (probe 4, cg01323777)**	−0.68	0.47	<0.0001	moderate
**KCNE3 (probe 1, cg18838431)**	−0.56	0.32	<0.0001	moderate
**KCNE3 (probe 2, cg02595219)**	−0.46	0.21	0.00	weak
**KCNE3 (probe 3, cg11775521)**	−0.54	0.3	<0.0001	moderate
**LOC100130872 (probe 1, cg14066993)**	−0.61	0.37	<0.0001	moderate
**LOC100130872 (probe 2, cg17232217)**	−0.59	0.35	<0.0001	moderate
**LOC100130872 (probe 3, cg27173148)**	−0.52	0.29	<0.0001	moderate
**MAMSTR (cg03127558)**	−0.41	0.17	0.00	weak
**MARCKSL1 (cg09072560)**	−0.38	0.15	0.01	weak
**NEFM (cg17078116)**	−0.43	0.19	0.00	weak
**SATB2 (cg10185119)**	−0.00125	0.000156	0.93	very weak
**SFRS13B (cg22363327)**	−0.35	0.12	0.01	weak
**SLC1A2 (probe 1, cg18494399)**	0.09	0.008	0.53	very weak
**SLC1A2 (probe 2, cg10159951)**	−0.14	0.02	0.33	very weak
**SLC1A2 (probe 3, cg21163960)**	−0.15	0.024	0.27	very weak
**TMEFF1 (cg22775000)**	−0.27	0.07	0.05	very weak
**TMEM97 (cg10342447)**	0.025	0.0006	0.86	very weak
**FOXL2 (cg01379240)**	−0.29	0.086	0.04	very weak
**TSPYL5 (cg00032205)**	−0.63	0.4	<0.0001	moderate

Supplementary Table 11: Common DMRs that are altered in STLMS compared to ULMS and STLMS compared to control tissue. See Supplementary Table 11

Supplementary Table 12: Common DMRs that are altered in ULMS compared to STLMS and ULMS compared to control tissue

Ref ID	UCSC_RefGene_ Name	UCSC_RefGene_ Accession	UCSC_ RefGene_ Group	Relation_to_ UCSC_CpG_ Island	Methylation status (ULMS compared to control tissue)	Methylation status (ULMS compared to STLMS)
cg02485642	MSLNL	NM_001025190	TSS200	Island	hypomethylated in ULMS compared to control	hypomethylated in ULMS compared to STLMS
cg08581018	MSLNL	NM_001025190	TSS200	Island	hypomethylated in ULMS compared to control	hypomethylated in ULMS compared to STLMS
cg03946671	TCEA2; TCEA2	NM_003195; NM_198723	TSS200; 5′UTR	Island	hypomethylated in ULMS compared to control	hypomethylated in ULMS compared to STLMS
cg24348495	TCEA2; TCEA2	NM_003195; NM_198723	TSS200; 5′UTR	Island	hypomethylated in ULMS compared to control	hypomethylated in ULMS compared to STLMS
cg05038268	KDM2A; KDM2A	NR_027473; NM_012308	TSS1500; TSS1500	Island	hypomethylated in ULMS compared to control	hypomethylated in ULMS compared to STLMS

cg21293464	KDM2A; KDM2A	NR_027473; NM_012308	TSS1500; TSS1500	Island	hypomethylated in ULMS compared to control	hypomethylated in ULMS compared to STLMS
cg08550839	FXYD1; FXYD7; FXYD1	NM_005031; NM_022006; NM_021902	Body; TSS1500; Body	Island	hypomethylated in ULMS compared to control	hypomethylated in ULMS compared to STLMS
cg14738670	FXYD1; FXYD7; FXYD1	NM_005031; NM_022006; NM_021902	Body; TSS1500; Body	Island	hypomethylated in ULMS compared to control	hypomethylated in ULMS compared to STLMS
cg10185119	SATB2	NM_015265	TSS200	Island	hypomethylated in ULMS compared to control	hypomethylated in ULMS compared to STLMS
cg10909152	SIVA1; SIVA1	NM_021709; NM_006427	TSS1500; TSS1500	Island	hypomethylated in ULMS compared to control	hypomethylated in ULMS compared to STLMS
cg12012524	CIRBP; C19orf23; CIRBP; CIRBP	NM_001280; NR_027271; NR_023313; NR_023312	TSS1500; Body; TSS1500; TSS1500	Island	hypomethylated in ULMS compared to control	hypomethylated in ULMS compared to STLMS
cg17336139	PRAME; PRAME; PRAME; PRAME; LOC648691; PRAME; PRAME; PRAME; PRAME	NM_206956; NM_006115; NM_206954; NM_006115; NR_027426; NM_206955; NM_206953; NM_206954; NM_206953	TSS200; 5′UTR; 1stExon; 1stExon; TSS200; TSS200; 5′UTR; 5′UTR; 1stExon	Island	hypomethylated in ULMS compared to control	hypomethylated in ULMS compared to STLMS
cg18725867	GDNF; GDNF; GDNF	NM_199231; NM_000514; NM_199234	5′UTR; 5′UTR; TSS200	Island	hypomethylated in ULMS compared to control	hypomethylated in ULMS compared to STLMS
cg20034372	LOC145845	NR_024264	TSS1500	Island	hypomethylated in ULMS compared to control	hypomethylated in ULMS compared to STLMS
cg24676071	ADCY1	NM_021116	TSS1500	Island	hypermethylated in ULMS compared to control	hypomethylated in ULMS compared to STLMS
